# Fertility preservation techniques in cervical carcinoma

**DOI:** 10.1097/MD.0000000000029163

**Published:** 2022-05-06

**Authors:** Erica Silvestris, Angelo Virgilio Paradiso, Carla Minoia, Antonella Daniele, Gennaro Cormio, Raffaele Tinelli, Stella D’Oronzo, Paola Cafforio, Vera Loizzi, Miriam Dellino

**Affiliations:** a Gynecologic Oncology Unit, IRCCS Istituto Tumori “Giovanni Paolo II” Bari, Italy; b Institutional BioBank, Experimental Oncology and Biobank Management Unit, IRCCS Istituto Tumori “Giovanni Paolo II,” Bari, Italy; c Unit of Hematology and Cell Therapy, Laboratory of Hematological Diagnostics and Cell Characterization, Bari, Italy; d Experimental Oncology, Center for Study of Heredo-Familial Tumors, IRCCS Istituto Tumori ”Giovanni Paolo II“ Bari, 70124 Bari, Italy; e Department of Biomedical Sciences and Human Oncology, Unit of Obstetrics and Gynecology, University of Bari Aldo Moro, Bari, Italy; f Department of Obstetrics and Gynecology, ”Valle d’Itria" Hospital, Martina Franca, Italy; g Department of Biomedical Sciences and Human Oncology, Section of Internal Medicine and Clinical Oncology, University of Bari Aldo Moro, Bari, Italy; h Department of Obstetrics and Gynecology, “San Paolo” Hospital, Bari, Italy; i Department of Biomedical Sciences and Human Oncology, Unit of Obstetrics and Gynecology, University of Bari Aldo Moro, Bari, Italy.

**Keywords:** cancer, cryopreservation, fertility preservation, in vitro fertilization, ovarian stem cells

## Abstract

The usefulness of this review is to highlight how a fertility preservation (FP) approach is currently feasible for patients diagnosed with uterine cervical cancer. To this regard, a fertility sparing surgery has just overcome its traditional limits, gained acceptance within the major gynecologic oncology societies thanks to the ability to identify the “ideal” candidates to this conservative treatment. On the other hand, the use of other FPs for oocyte and ovarian cortex cryopreservation is still extremely debated. In fact, the existing risk of tumor spreading during oocyte retrieval necessary for oocyte cryostorage for patients’ candidates for neo-adjuvant therapy, as well as the potential hazard of cancer cell dissemination after ovarian tissue replacement in cases of non-squamous type cervical carcinomas should not be underestimated. Therefore, in consideration of the encountered limitations and the need to ensure adequate reproductive health for young uterine cervical cancer survivors, translational research regarding the FP has progressively collected innovative insights into the employment of stemness technology. In this context, the property of ovarian stem cells obtained from the ovarian cortex to generate functional oocytes in women could represent a promising therapeutic alternative to the current procedures for a novel and safer FP approach in cancer survivors.

## 1 Introduction

Uterine cervical cancer (UCC) is the fourth most common malignancy in women worldwide. In 2018, the International Agency for Research on Cancer (IARC) reported 569,000 new patients with a mortality as high as 311,000 cases^[1]^ and a prevalence of 35 to 44 years aged. However, this cancer also affects women older than 65 years who are generally less susceptible to preventive screening.^[2]^ Pathogenesis of UCC is mainly related to infection by human papillomavirus (HPV), a DNA virus belonging to the Papillomaviridae family, and both HPV-16 and HPV-18 are apparently involved in up to 70% of cases.^[3,4]^ These high-risk HPV strains are able to trigger the primary carcinogenic effect and encourage tumor promotion through the expression of several viral oncoproteins that can interfere with and deregulate major cell activities (cell cycle, proliferation, and apoptosis).^[5]^ However, although HPV infection is widespread and is the main etiological factor in the process of carcinogenesis, it is not always detectable in all patients affected by UCC and, at the same time, does not certainly lead to cancer in a minority of subjects. Thus, since HPV is not detectable in every UCC patient, it has been postulated that other concomitant factors such as herpes simplex virus (HSV) type 2 infection, smoking attitude, improper nutrition, and oral contraceptive consumption, as well as low economic status and poor personal and sexual hygiene, may contribute to the development of this neoplasm.^[3]^Although UCC is diagnosed at various clinical stages, a consistent percentage of patients are successfully treated with conventional strategies, including radical surgery, radio- and chemotherapy, and by new drugs acting through molecular targeting of cancer cells. However, the clinical outcome is variable, with a range of 20% to 70% of relapse for the early and advanced stages, respectively,^[6]^ and an average 5-year survival is observed in about 66% of UCC patients at all ages.^[7]^ On the other hand, the survival rate is apparently related not only to the clinical stage and tumor pattern at the time of diagnosis (from 92% for early stage invasive UCC to 56% for loco-regional extension, up to 17% for metastatic variants) but also to the race, ethnicity, and age of patients.^[2]^ Currently, two different types of diagnostic tests for UCC are recognized as Pap smear and HPV tests, respectively. The first is diagnostic for both precancerous and cancerous cellular lesions and is useful for planning relative treatments. On the contrary, the HPV test is the gold standard approach to reveal HPV DNA or RNA, thus allowing HPV type identification. Despite the implementation of social screening and treatment protocols for UCC leading to survival improvement, the therapeutic approach to this disease, which involves surgery and radio- and chemotherapy, may dramatically impair female fertility with a detrimental effect on ovarian function, resulting in early ovarian insufficiency and premature menopause. Such reproductive failure due to cancer-related imbalance inevitably leads to worsened quality of life, particularly in patients within their fertile age. In this context, several fertility preservation (FP) technologies, such as oocyte retrieval and cryopreservation, as well as autologous ovarian cortex cryopreservation and transplantation currently achieved before starting neoadjuvant systemic chemotherapy, surgery, or radiation treatments, provide a suitable potentiality in preserving the ovarian endocrine function in young cervical cancer patients.^[8]^ Other techniques also consider the application of stemness technologies that adopt ovarian stem cells (OSCs).^[9]^ As already proven in other fields of regenerative medicine, adult stem cells are capable of reconstituting tissues and OSCs have been described to differentiate in vitro in oogones, namely oocyte like cells (OLCs), which in animal models are suitable for recovering fertility after pharmacological sterilization. Once this technology is improved and adopted in treating infertility in humans, the availability of OLCs from each UCC patient will provide other opportunities to restore the oocyte reserve suitable for conception after cancer healing. Here, the leading objective of this review is to focus the infertility risk related to UCC treatment and on the other hand the therapeutic strategies currently available for FP in young female cancer patients.

## 2 Methods

The systematic review will be performed following the guidelines of the Preferred Reporting Items for Systematic Review and Meta-Analysis Protocols (PRISMA-P) guidelines.

Ethical approval is unnecessary because this is a literature-based study.

### 2.1 Types of participants

We considered patients that are successfully treated for UCC with conventional strategies, including conservative surgery, radio- and chemotherapy, and by new drugs acting through molecular targeting of cancer cells irrespective of their sex, age, severity, and disease duration.

### 2.2 Types of interventions

A collection of literature was performed in women of childbearing potential with UCC, both of those more standardized techniques and of the most innovative approaches such as the use of stem cells.

### 2.3 Data sources and search methods

#### 2.3.1 Electronic searches

Relevant studies searched in the following electronic databases: PubMed, the Cochrane Library, Embase, Web of Science, and Medline databases. The following search terms used: cancer; cryopreservation; FP; in vitro fertilization; OSCs.

### 2.4 Searching for other resources

Additionally, the international clinical trials registry platform, dissertation, and gray literature also searched to identify systematic reviews related to UCC and FP. The relevant conference papers, journals retrieved manually.

### 2.5 Data collection and analysis

Selection of studies. Two researchers were independently discussing and determine research selection process according to the criteria. We removed the duplicated data and screen records by title and abstract and the full article.

### 2.6 Data extraction and management

Titles and/or abstracts of studies retrieved using the search strategy, and those from additional sources, were screened independently by two review authors to identify studies that potentially meet the aims of this non-systematic review. The full text of these potentially eligible articles was retrieved and independently assessed for eligibility by another two review team members. Any disagreement between them over the eligibility of articles was resolved through discussion with a third (external) collaborator. Two authors independently extracted data from articles about study features and included populations, type of intervention and outcomes. Any discrepancies were identified and resolved through discussion (with a third external collaborator where necessary). Due to the nature of the findings, we opted for a narrative synthesis of the results from selected articles. Despite the therapeutic approaches to this cancer showing increasing effectiveness, probably as effects of personalized treatments, the conventional and novel approaches to treat UCC include the risk of transient or permanently exhausted ovarian reserve, and personalized female FP strategies are adopted based on previous experience acquired in similar programs in other cancers, which currently provide encouraging results so far. Besides these well-experienced procedures, which reconstitute fertility in a large proportion of patients with UCC, the possibility of applying novel stemness technologies, including neo-oogenesis by OSCs, needs to be further investigated.

## 3 Cervical carcinoma treatments and infertility risk

**Figure 1:**
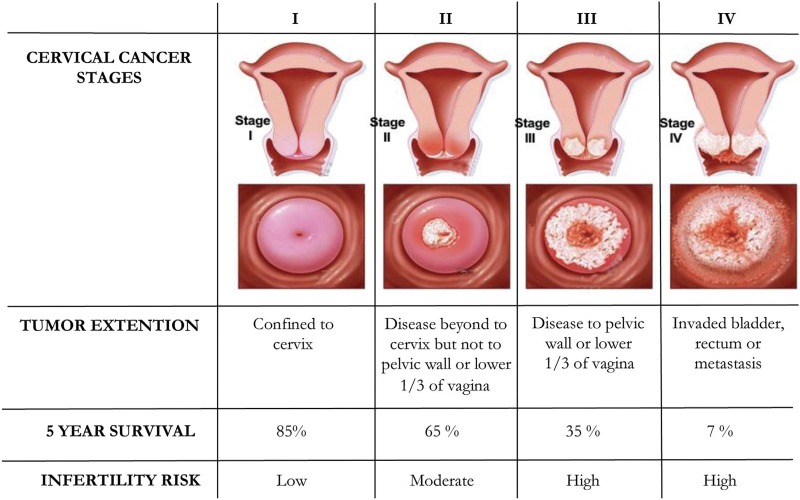
Staging of cervical cancer and infertility risk disease related.

UCC is a highly heterogeneous disorder whose prognosis depends on both growth pattern and diffusion, as in most cancers. In fact, besides the mentioned prognostic factors, connective tissue invasion between the cervix and parametrium, exophytic development, and lymph node metastatic involvement can seriously worsen the disease prognosis.^[10]^ Additional prognostic factors for tumor relapse have also been suggested. Recently, in a retrospective study, Koulis et al reported the role of several hematologic parameters, such as anemia, leukocytosis, neutrophil-to-lymphocyte ratio, and thrombocytosis as potential indicators of worsening outcomes in UCC patients during their treatment.^[11]^ Other authors stated that a peculiar molecular pattern of UCC, including the expression of apoptotic molecules and genomic derangements, provides additional information for poor prognosis. In the early stages of HPV infection, the virus is suspected to modify the expression of several genes, including the receptor subunit 1 of both IFNα and-β (IFNαR1), epithelial membrane protein 1 (EMP1), and interleukin 1 receptor antagonist (IL1RN) capable of activating the UCC escape from the host immune surveillance, thus favoring cancer progression.^[12]^ A similar effect is apparently induced by HPV in down-regulating the type 2 receptor of interleukin1 (IL1R2),^[13]^ whereas other investigators have shown that the Hippo-Yap pathway plays a primary role in cervical epithelium carcinogenesis through the interaction of the Yes-associated protein (YAP1) with the HPV E6 oncoprotein.^[14]^In line with the International Federation of Gynecologists and Obstetricians (FIGO) 2018 criteria,^[15]^ the UCC treatment is mainly related to the stage of the disease, which primarily includes the tumor size and its spread to nearby organs as well as metastases, but it is also adjusted for patient age and comorbidity.^[16]^ Generally, there are no standard medical therapies in Ib-IIa early stages since both radical surgery including hysterectomy/trachelectomy with lymph node dissection and radiotherapy have been proven to be equally effective, although they differ in related morbidity and complications. However, in several oncologic institutions, the surgical approach is combined with a subsequent radiation protocol with or without chemotherapy using a platinum-based regimen, while in other centers, the primary treatment recommends chemo-radiation protocols. Finally, in advanced stages (III-IVa), concomitant chemoradiotherapy is the standard treatment in relation to the proven capability to provide improved disease-free, progression-free, and overall survival periods.^[17]^ Besides the detrimental effects on the ovarian reserve as effects of pelvic surgery, particularly radiotherapy, most chemotherapy agents adopted in UCC treatment are known for their potential in affecting the viability of oocytes with a high risk of infertility (Fig. 1).

Currently, there are no data clarifying the relative percentage of infertility risk for each UCC stage.

In particular, platinum-containing compounds such as cisplatin and carboplatin are commonly used despite their gonadotoxicity related to well-known DNA derangements,^[18]^ although other molecular detrimental mechanisms (oxidative stress induction, modulation of calcium signaling, and deregulatory effects on intracellular molecular pathways) are directly promoted. The loss of the ovarian primordial follicles by a DNA-damage-induced apoptosis mechanism related to p53 activation is thought to be the major restraining effect of platinum derivatives.^[19]^ Paclitaxel, a taxol byproduct, is also widely used in the treatment of gynecologic malignancies and has been proven to significantly decrease the maturation of antral follicles, as well as the viability of corpora lutea resulting in follicular atresia, as shown in mouse models.^[20]^ Furthermore, bevacizumab is an anti-angiogenic agent commonly used to treat relapsing and/or metastatic cervical cancer. Its combination with other drugs or by itself, is dangerous for the oocyte maturation due to Hypoxemia induced in ovaries,^[21,22]^ while there are not available data on the potential toxicity on female reproductive organs by topotecan, a further chemoterapic drug frequently used in advanced gynecologic neoplasms.^[23]^ These cytotoxic drugs, currently employed in advanced and metastatic UCC, are able to guarantee beneficial effects in approximately 30% of patients, ensuring up to 10 months of overall survival. Thus, novel therapeutic alternatives using molecular targeting agents have been intensively investigated for improved responses.^[24]^ To this end, particular interest has been recently devoted to exploring the molecular aberrations in UCC, and major therapeutic improvements include antibody-drug conjugates, vaccines, and immune checkpoint inhibitors (ICIs). Previous studies have reported an association between the expression of PD-1 (programmed cell death 1) and PD-L1 on cervical cancer infiltrating T cells, with high-risk HPV positivity and increasing cancer grade. The expression of PD-1 by a large fraction of infiltrating CD8T cells in cervical cancer suggests that blocking PD-1 by human monoclonal antibodies has therapeutic potential.^[24]^ However, despite the encouraging data concerning the efficacy of ICIs in UCC,^[24]^ the effects of these agents on oogenesis and in general on the pathophysiology of female fertility are presently unclear,^[25]^ and their combination with therapeutic vaccines needs to be properly investigated in relation to the potential alterations in the immune system steady state, resulting in immune tolerance derangement and autoimmune development.^[26]^ During anti-cancer treatments, the incidence of iatrogenic ovarian failure depends on several aspects, including the patient's age and the therapeutic program to defeat the tumor. Nearly 40% of women diagnosed with stage I UCC are younger than 40 years^[27]^ thus emphasizing that this cancer significantly recurs at an early stage in females during their reproductive period and that its treatment is strongly related to the risk of infertility. For this reason, FP in anti-cancer therapeutic options in young patients must play a pivotal role. In young UCC patients, ovarian failure related to cancer treatment may dramatically affect the quality of life of patients after cancer healing. Beyond cancer-related infertility and sexual dysfunction, patients also experience negative psychosocial effects such as depression, stress, and anxiety.^[28]^ Furthermore, exhausted ovarian function results in lower hormone bioavailability, leading to premature menopause, which typically occurs with vasomotor symptoms and genitourinary disorders^[29]^ in 68%, while early hypoestrogenism may drive cardiovascular illnesses in 54%^[30]^ and osteoporosis due to cancer treatment-induced bone loss with increased risk of bone fractures in 67%.^[31,32]^ However, although novel therapeutic options for FP have recently endorsed several successful pregnancies in young UCC survivors,^[33]^ clinicians do not regularly focus attention on this topic.

## 4 FP strategies in cervical cancer

The topic of FP in women with UCC actually represents an interesting and developing issue, mainly in relation to significant advancements in the treatment of this disease.^[34,35]^ The improvement of primary and secondary prevention through the introduction of HPV screening programs and vaccination has led to the early diagnosis of UCC in order to prevent its subclinical evolution; however, a large proportion of this cancer is actually diagnosed in reproductive-aged women who have not yet realized their motherhood desires. Therefore, in these cases, the American Society of Clinical Oncology (ASCO) guidelines,^[36]^ recommend extensive counseling with a structured oncofertility team, including gynecologic surgeons, oncologists, and reproductive specialists, to explain the potential drawbacks of cancer treatment and related infertility risk.^[37]^ Therefore, several aspects of the therapeutic approach to the disease, such as age at diagnosis, parity, desire of motherhood, ovarian reserve status, and available time from diagnosis to the beginning of the cancer treatment, need to be assessed in addition to tumor stage, malignancy grading, and prognosis. In UCC, a few FP strategies are currently available, as reported in Table 1.

**Table 1:** Staging of cervical cancer, treatment and related fertility preservation approach.

UCC Stage	Treatment	Fertility preservation approach
Early disease		
IA1	Conization	Conization
IA2, IB, IIA	Combined radiation with brachytherapy and radical hysterectomy with lymphadenectomy	Fertility sparing surgery (Radical vaginal trachelectomy with pelvic lymph node dissection)
	Cisplatin-based chemotherapy with radiation (if high-risk features as positive lymph nodes, surgical margins and/or parametria	
Advanced disease		
IIB, III, IVA	Cisplatin-based chemotherapy with radiation	Ovarian suppression with GnRHa before/during CHT Ovarian transposition before RT Oocyte cryopreservation before neo-adjuvant CHT or combined CHT-RT Ovarian cortex cryopreservation In vitro differentiation of OSCs
Metastatic disease		
IVB, recurrent cancer	Cisplatin as palliative, radiation therapy for control of bleeding and pain and systemic chemotherapy for disseminated disease	Although it is ethically inadvisable, it is nonetheless applicable a gestional surrogacy

Due to its epidemiological characteristics, cervical cancer is the most represented and candidate for fertility-sparing surgery in early disease, contrary to the advanced and metastatic stages for which other FP approaches are advisable.^[38,39]^

These include well-established ovarian suppression with gonadotropin-releasing hormone agonists (GnRHa), as well as other procedures such as fertility-sparing surgery, ovarian transposition, ovarian cortex cryopreservation, and oocyte cryopreservation for later assisted reproductive techniques (ART). Here, we summarize these FP practices, which are mandatory to be completed in a short time before starting the anti-cancer treatment against UCC.

### 4.1 Ovarian suppression with GnRHa (Gonadotropin-releasing hormone agonists)

The inhibition of oocyte maturation during or just before a chemotherapy regimen with a concomitant treatment with gonadotropin-releasing hormone agonist (GnRHa), such as goserelin, triptorelin, buserelin, and leuprolide represents a well-established FP approach in cancer patients. Data from comprehensive literature highlight that, when other FP methods are not feasible, GnRHa administration may be offered to reduce chemotherapy-induced ovarian toxicity.^[36]^ However, despite the fact that GnRHa utilization in FP programs is still debated, this therapeutic approach could equally be considered applicable for urgent cases.^[40]^

### 4.2 Fertility sparing surgery

Among gynecological tumors, UCC is the major candidate for conservative surgery, mainly because of the available prevention programs, which allow early diagnosis in young patients with possible time-related surgical management. Even if the standard treatment for patients with early stage UCC includes hysterectomy and pelvic lymphadenectomy,^[41]^ in women interested in future pregnancies and with limited disease, a conservative surgical approach is recommended. In particular, considering that the UCC typically involves lymph nodes with consequent extension of the disease, a careful evaluation of their consistency during surgery is necessary to assess the feasibility of the proper FP-sparing surgical procedure. With respect to stage IA1 UCC (carcinoma strictly confined to the cervix; maximum depth of invasion <5 mm; stromal invasion <3 mm in depth) without lymphovascular space invasion (LVSI), the risk of lymph node involvement is <1%.^[42]^ Therefore, the loop electrosurgical excision procedure or cold knife conization followed by endocervical curettage with negative surgical margins, can provide a definite option with a minimal relapse recurrence of <0.5%^[42]^ with a 99% of 5-year survival rate which is superior to extended hysterectomy (98%).^[43]^ However, in the case of IA1 with positive LVSI, the risk of clinical recurrence may increase up to 9%, and radical trachelectomy, namely, cervix excision with surrounding parametria proximal to the isthmus and subsequent uterus-vagina suture, or simple trachelectomy, which excludes parametria excision,^[44]^ with additional pelvic lymph nodes and sentinel node mapping, have been suggested.^[45]^ Moreover, from stages IA2 UCC (cancer strictly confined to the cervix; maximum depth of invasion <5 mm; stromal invasion ≥3 mm and <5 mm in depth) to IB1 (tumor <2 cm in major dimension), lymph node positivity increases from 5–7% to up to 16%. In stage IA2 with negative LVSI, conization alone was considered curative. Instead, in patients with stage IA2 with positive LVSI, after nodal involvement exclusion, radical or simple trachelectomy additional thorough pelvic lymph node dissection and sentinel node mapping are stringently suggested.^[46]^ On the other hand, according to the European Society of Gynaecological Oncology (ESGO) guidelines, in the case of young women with IB1 stage and negative nodes,^[47]^ a radical trachelectomy is also recommended. Nowadays, considering the lower rate of a parametrial implication (range between 0.4% and 0.6%),^[48]^ a less radical approach such as simple trachelectomy or conization is to be evaluated, in order to lower both surgical and obstetric morbidity.^[49]^ Finally, for tumors between 2 and 4 cm (IB2), neoadjuvant chemotherapy followed by conization or the simple/radical trachelectomy could also represent a feasible FP strategy^[50]^ with outcomes similar to standard management (recurrence rate 8.5%).^[51]^ However, conservative surgical treatment for patients with IB2 stage should be offered only in selected patients,^[52]^ since it is still considered an experimental approach.

### 4.3 Ovarian transposition

Although randomized controlled trials on reproductive outcomes through ovarian transposition are limited, the success rate of this FP procedure is ∼90%. Therefore, ovarian transposition is considered an effective FP technique and is obtained by distancing the ovaries in the abdomen, namely by removing their allocation from the irradiation field.^[53,54]^ However, for the novel place of the transposed ovary, this procedure disables in woman the possibility of a transvaginal oocyte retrieval in future programs of in vitro fertilization and several clinicians are prone to suggest to UCC patients candidate to chemoradiation a “combined approach,” namely the transposition of a selected ovary and the cryopreservation of the other one.^[55]^ On the other hand, high-precision modern radiation therapy methods such as MRI-guided brachytherapy, it is now possible to selectively target the cervix and exclude the uterine corpus from radiation damage.^[24]^

### 4.4 Oocyte cryopreservation

ASCO and the European Society for Medical Oncology (ESMO) endorse both oocyte and embryo cryopreservation before anti-cancer treatment to guarantee motherhood in female cancer survivors.^[36]^ At present, the predictability of oocyte cryopreservation success is related to the viability of oocytes recovered and stored after controlled ovarian stimulation (COS) by gonadotropin. However, several drawbacks prevent the suitability of this procedure, particularly in UCC patients requiring urgent anti-cancer treatments, including neoadjuvant chemotherapy protocols. In this case, there is probably insufficient time to induce COS based on the time required for oocyte maturation.^[42,56]^ On the other hand, oocyte cryopreservation needs to be regulated in relation to the patient and the treatment to be addressed^[57]^; for instance, for patient candidates to undergo surgery, the oocyte pick-up before surgery is frequently effective in recruiting a suitable number of oocytes, although the procedure can potentially cause cancer spread. In conclusion, oocyte cryopreservation should be completed before neo-adjuvant chemotherapy or combined chemoradiation and after informing patients about the risks of iatrogenic ovarian/uterus injury and spread of cancer during oocyte pick-up.

### 4.5 Ovarian cortex cryopreservation

In patients urgently requiring anti-cancer treatments at risk of genotoxicity, ovarian tissue cryopreservation and auto-transplantation have been proposed.^[58,59]^ This practice could be employed independently from both hormone stimulation and menstrual cycle phases, and implies ovarian sampling by either laparoscopic or laparotomic pelvic access followed by orthotopic or heterotopic autologous reimplantation after cancer healing. However, the procedure includes the potential risk of replanting tumor cells along with ovarian tissue transplantation. Several authors have reported an increased incidence of ovarian involvement in UCC, particularly in the non-squamous type and in patients with FIGO 2018 stage IIB cervical adenocarcinoma. Consequently, in young women with UCC, the istotype and stage evaluation should be considered during oncofertility counseling in order to correctly suggest an appropriate and personalized FP method for each patient.^[60]^

## 5 Fertility and obstetric outcomes after UCC treatment

In relation to the cancer stage and the established surgery, it is pivotal for each UCC patient to receive accurate information concerning their chance to conceive after treatment as well as on the potential complications related to the FP procedures, which primarily include preterm and premature delivery as a result of the restricted anatomical support due to cervical exeresis.^[61]^ The fertility rate following cervical surgical excision in stage I UCC patients ranges around 55%. In particular, simple trachelectomy and radical vaginal trachelectomy are associated with a similar conception rate. On the other hand, abdominal laparoscopic radical trachelectomy further reduces the fertility rate to 40%.^[61]^ Similarly, the pregnancy rate was higher in patients who underwent vaginal or minimally invasive radical trachelectomy than in those who underwent laparotomic surgery.^[62]^ Moreover, a large review of literature highlights that patient with UCC, treated with a distinctive fertility-sparing surgery approach, reported overall fertility, live birth, and prematurity rates of 55%, 70%, and 38%, respectively.^[62]^ Consequently, all patients who became pregnant after UCC should be informed about the obstetric risks and precautionary referral to a gynecological and obstetrical center equipped with neonatal intensive care. On the other hand, patients treated with either neo-adjuvant (NACTH) or adjuvant chemotherapy protocols before conization or simply trachelectomy are at a lower risk of premature delivery.^[63]^ Undoubtedly, NACTH allows a less extensive surgery with a minor impact on the length of the cervix/uterine isthmus, although it has a potential gonadotoxic effect.^[55]^ Therefore, in patients undergoing fertility-sparing surgery, oocyte cryopreservation before gonadotoxic treatments could be proposed, although the risks of malignant cell diffusion during oocyte pick-up are realistic.^[56,64]^ On the other hand, in UCC requiring extensive surgery, the potential treatment for fertility restoration includes uterus transplantation or gestational surrogacy practice.^[65]^ In this regard, it has been described that once oocytes have been recruited and cryopreserved from patients subjected to hysterectomy, they could be used in surrogate pregnancy to restore oogenesis resulting in pregnancy.^[65]^ However, successful surrogate pregnancies have been reported only in a few young UCC women who underwent first hysterectomy and radiotherapy with ovarian transposition and later transabdominally oocyte retrieval.^[65]^ On the contrary, results concerning successful pregnancy after ovarian cortex cryopreservation and reimplantation are still lacking. A potential alternative to the conventional FP methods includes the ovarian tissue cryopreservation, which in case of non-squamous UCC involves a potential higher risk of cancer cell spread following the ovarian tissue reimplantation. In fact, literature data, report in stage IIb, a higher incidence of ovarian FP procedure-related metastases; thus, ovarian tissue cryopreservation in these patients is not recommended.^[66]^ In conclusion, the available evidence on the effectiveness and safety of oocyte storage and ovarian tissue cryopreservation in women with UCC is limited, and alternative risk-free strategies are needed for FP in young patients with this malignancy.

## 6 In search of safe procedures for FP in cervical carcinoma

FP programs in cancer patients have recently attracted great interest from clinicians and FP teams in relation to stemness technology in the field of regenerative medicine. In this context, despite the limitations concerning oocyte retrieval and ovarian cortex cryopreservation in UCC patients, a novel application of OSCs from the ovarian cortex has gained increasing interest.^[67]^ In fact, when these cells are grown in vitro under appropriate conditions, they are able to generate functional oocytes undergoing a final cell differentiation stage in oocyte-like cells (OLCs) with morphological and molecular patterns similar to well-differentiated oocytes.^[67]^ To support this evidence, additional data from recent literature also show that OSCs isolated from both young and post-menopausal women grow in short-term cultures as OLCs and express typical markers of final oocyte maturation as SYCP3 and GDF9,^[9]^ thus proving the suitability of these cells for further investigation in future programs of fertility reconstitution in female patients at risk of infertility for anti-cancer treatments. On the other hand, the discovery of OSCs may open new avenues for the general treatment of infertility in women with poor ovarian reserve and represents an overcoming of the “mammalian fixed ovarian reserve” dogma in reproductive sciences. This long-held belief, according to which, in postnatal mammalian ovaries of most species, no renewable germinal OSCs are available and, thus, supporting the hypothesis that during the lifetime, a numerically fixed pool of oocytes is committed to fertility.^[68]^ Therefore, in young female UCC patients, the possibility of developing in vitro progeny of OLCs from single OSCs, selecting good-quality eggs to be frozen and subsequently utilized in FP represents significant advantages in terms of safety of the practice.^[60]^ In fact, with respect to the traditional hyperstimulation procedure necessary for oocyte recruitment, the manageable recovery of ovarian cortex fragments will render the application of this stemness technology to FP and infertility treatment in the future. However, although additional studies are required for future OSC application, when compared to conventional FP procedures, the ability of these cells to reestablish oogenesis offers an additional advantage in terms of hormonal balance since their reimplantation in anovulatory ovaries.^[60]^

## 7 Conclusion

UCC is the fourth most common malignancy in women worldwide, both in pre- and post-menopausal women. Recent advances in the past few years in screening and early diagnosis of this tumor have guaranteed a significant improvement in the expectancy and quality of life of UCC cancer survivors through modern surgical and anti-cancer treatments. However, despite these practices are mainly adopted, they are not entirely safe for female reproductive function and may affect fertility in young patients planning maternity programs after cancer healing. Despite the therapeutic approaches to this cancer showing increasing effectiveness, probably as effects of personalized treatments, the conventional and novel approaches to treat UCC include the risk of transient or permanently exhausted ovarian reserve, and personalized female FP strategies are adopted based on previous experience acquired in similar programs in other cancers, which currently provide encouraging results so far. They generally include fertility-sparing surgery options as well as oocyte and ovarian cortex cryopreservation, whose adoption for each single patient needs to be assessed by a multidisciplinary reproductive team, in order to select the most suitable procedure. Besides these well-experienced procedures, which reconstitute fertility in a large proportion of patients with UCC, the possibility of applying novel stemness technologies, including neo-oogenesis by OSCs, needs to be further investigated. This innovative application of regenerative medicine in infertility offers the advantage of selecting the eggs to be fertilized and is safe from additional oncogenic risk related to hormone hyperstimulation usually adopted in cancer patients undergoing preparation for MFG for oocyte recruitment. However, intensive investigation is still required before translating this technology to FP in patients with cancer.

## Author contributions

Conceptualization, E.S., M.D, S.D.; Data curation, G.G. and R.T.; Original draft writing: E.S., M.D. S.D., P.C. Review and editing, S.D., A.V.P., G.C. All authors have read and agreed to the published version of the manuscript.

**Conceptualization:** Erica Silvestris, Gennaro Cormio, Miriam Dellino.

**Data curation:** Carla Minoia, Erica Silvestris, Miriam Dellino, Vera Loizzi.

**Formal analysis:** Carla Minoia, Erica Silvestris, Miriam Dellino, Stella D’Oronzo.

**Formal analysis:** Miriam Dellino.

**Funding acquisition:** Angelo Paradiso.

**Investigation:** Miriam Dellino, Raffele Tinelli.

**Methodology:** Miriam Dellino.

**Project administration:** Miriam Dellino.

**Resources:** Paola Cafforio.

**Writing – review & editing:** Antonella Daniele.
